# Risk Factors for Severe Influenza A–Related Pneumonia in Adult Cohort, Mexico, 2013–14

**DOI:** 10.3201/eid2009.140115

**Published:** 2014-09

**Authors:** Alejandro Gómez-Gómez, Martin Magaña-Aquino, Sofía Bernal-Silva, Javier Araujo-Meléndez, Andreu Comas-García, Emma Alonso-Zúñiga, Eliana Torres-Torres, Daniel E. Noyola

**Affiliations:** Hospital Central “Dr. Ignacio Morones Prieto,” San Luis Potosí, Mexico (A. Gómez-Gómez, M. Magaña-Aquino, J. Araujo-Meléndez); Instituto Nacional de Salud Pública, Cuernavaca, Mexico (A. Comas-García);; Universidad Autónoma de San Luis Potosí, San Luis Potosí (A. Gómez-Gómez, M. Magaña-Aquino, S. Bernal-Silva, J. Araujo-Meléndez, E. Alonso-Zúñiga, E. Torres-Torres, D. E. Noyola)

**Keywords:** Influenza, A(H1N1)pdm09, viruses, vaccine, pandemic, pneumonia, viral pneumonia, diabetes, intensive care, obesity, overweight, body mass index, BMI, ventilator, zoonoses

## Abstract

During the 2013–14 influenza season, we assessed characteristics of 102 adults with suspected influenza pneumonia in a hospital in Mexico; most were unvaccinated. More comorbidities and severity of illness were found than for patients admitted during the 2009–10 influenza pandemic. Vaccination policies should focus on risk factors.

During 2009, Mexico reported 3 outbreaks of influenza A(H1N1)pdm09 infection (*1*); during the 2011–12 winter, a fourth pandemic wave of illness attributed to the virus was reported (*2*). Many patients had severe pneumonia related to influenza A(H1N1)pdm09 virus; the death rate was higher in Mexico than in most countries (*3*–*5*).

Beginning December 5, 2013, admissions of adults with acute respiratory infections increased at Hospital Central “Dr Ignacio Morones Prieto” (Hospital Central), in San Luis Potosí, Mexico; 102 persons were admitted with suspected influenza pneumonia. A high proportion of patients required mechanical ventilation (MV) and intensive care. The number of acute respiratory infection–associated admissions of young children and older adults did not increase. This pattern, occurring nearly 5 years after the initial influenza A(H1N1)pdm09 outbreak, resembles that of the initial outbreak in San Luis Potosí in April 2009 (*6*). This raises questions regarding possible changes in the viral strain or the presence of large numbers of susceptible persons not exposed to, or vaccinated against, this virus. We analyzed characteristics of 102 patients with confirmed or suspected influenza pneumonia admitted during a 2-month period and compared them with those of 100 patients with confirmed A(H1N1)pdm09 infection hospitalized during the 2009–10 pandemic.

## The Study

During December 5, 2013–February 7, 2014, 102 patients, 17–79 years of age, were admitted to Hospital Central with acute onset of cough or rhinorrhea plus dyspnea, fever, and radiographic evidence of pneumonia and were considered to have possible influenza pneumonia. We compared data for the 2013–14 cohort with data from patients 15–71 years of age with confirmed influenza A(H1N1)pdm09 infection admitted to Hospital Central during the 2009–10 pandemic (*7*). Patients were evaluated according to a clinical questionnaire (*6*,*7*); blood samples (laboratory tests and cultures), sputum samples or tracheal aspirates (Gram stain and culture), and chest radiographs were also evaluated. Nasopharyngeal or tracheal samples were analyzed to detect influenza virus by using real-time reverse transcription PCR (Laboratorio Estatal de Salud Pública, Servicios de Salud del Estado de San Luis Potosí) or sequence-specific primer PCR (Universidad Autónoma de San Luis Potosí) with previously reported primers (*8*). Empiric treatment (ceftriaxone and clarithromycin or ceftriaxone and levofloxacin) was administered, and a course of oseltamivir was started within 6 hours after admission at doses of 75–150 mg 2× daily.

Influenza A(H1N1)pdm09 virus was detected in 47 patients and influenza A (not subtyped) in 8 patients; samples were not submitted for virologic testing for 8 patients. In 28 (71.8%) of 39 patients with negative results by real-time and sequence-specific primer reverse transcription PCR, samples were collected >5 days after onset of symptoms or patients had received oseltamivir during >1 day at the time of sample collection; therefore, test results for those patients were not conclusive, and the patients were included in the cohort. Signs and symptoms of the remaining 11 patients with negative results and of the 8 untested patients were suggestive of influenza infection; therefore, they were included in the cohort. 

Mean age of the 102 patients was 44.6 years; 52 (51%) were female, and 82 (80.4%) had risk factors for complications. The most common concurrent health conditions were obesity (63[62%]) and diabetes mellitus (23 [23%]). Eight (8%) patients had been vaccinated for 2013–14 seasonal influenza. Radiologic manifestations were bilateral ground glass opacities and consolidations. Most patients had severe illness; mean partial pressure arterial oxygen and fraction of inspired oxygen ratio on admission was 175.6 (reference value 400; SD 74.4); 52 (50.9%) patients required MV.

Demographic and clinical characteristics of patients are shown in Table 1. The 52 patients receiving MV were more likely to be obese (body mass index [BMI] >30) than those who did not require it (76.5% vs. 46.9%, respectively; p = 0.002). Lactate dehydrogenase concentrations (406.8 mg/dL vs. 694.6 mg/dL; p = 0.006) and C-reactive protein level (12.9 mg/dL vs. 19.7 mg/dL; p = 0.001) were higher in patients who required MV. 

**Table 1:** Characteristics of 102 patients with severe pneumonia who required mechanical ventilation compared with patients who did not require mechanical ventilation, San Luis Potosi, Mexico, 2013–14*

Characteristic	Without mechanical ventilation (n = 50)	With mechanical ventilation (n = 52)	p value
Age, y, mean (SD)	42.8 (13.6)	46.2 (13.3)	
Gender, no. (%)			
M	25 (50)	25 (48.1%)	0.21
F	25 (50)	27 (51.9%)	0.85
Presence of ≥1 underlying conditions, no. (%)	38 (76)	44 (84.6%)	0.27
Obesity (body mass index >30) †	23 (46.9)	39 (76.5%)	0.002
Diabetes mellitus	12 (24)	11 (21.2%)	0.73
Immunosuppression‡	4 (8)	5 (9.6%)	1.0
Asthma-COPD-bronchiectasis	8 (16)	1 (1.9%)	0.01
Other §	9 (18)	3 (5.8%)	0.06
Underlying disorders, excluding obesity, no. (%)	27 (54)	21 (40.4%)	0.17
Influenza vaccination during most recent season, no. (%)	8 (16)	0	0.002
Signs and symptoms, no. (%)			
Fever	49 (98)	52 (100%)	0.49
Headache	45 (90)	49 (94.2%)	0.48
Cough	50 (100)	52 (100%)	NA
Myalgias	46 (92)	46 (88.5%)	0.74
Dyspnea	50 (100)	52 (100%)	NA
Blood-streaked sputum	14 (28)	15 (28.8%)	0.92
Diarrhea	9 (18)	6 (11.5%)	0.36
Clinical findings, mean (SD)			
Body mass index*	29.6 (4.8)	31.9 (3.9)	0.01
Respiratory rate, bpm	26.4 (3.3)	30.3 (5)	<0.001
Cardiac rate, bpm	102.6 (12.4)	109.2 (16.9)	0.03
Mean blood pressure, mmHg	80.6 (11.3)	74.2 (14.6)	0.01
Temperature, °C	38.5 (0.5)	38.5 (0.5)	0.42
PaO_2_/FiO_2_	236 (49.2)	119.8 (43.8)	<0.001
Laboratory findings, mean (SD)			
Total WBCs, x10^3^/μL†	8.1 (4.3)	7.4 (3.9)	0.39
Lymphocytes, x10^3^/μL†	1.2 (0.75)	0.84 (0.64)	0.01
Platelet count, x10^3^/μL†	223.5 (93.1)	212.4 (125.4)	0.62
C-reactive protein, mg/dL†	12.9 (8.8)	19.7 (11.6)	0.001
Lactate dehydrogenase, U/L #	406.8 (416.6)	694.6 (547.4)	0.006
CPK, U/L **	349.5 (760.1)	575.9 (544.4)	0.1
Symptom onset			
Duration of symptoms before dyspnea, d, mean (SD)	2.8 (2.8)	3.1 (3.3)	0.67
Dyspnea duration before admission, d, mean (SD)	3.9 (2.8)	3.7 (2.7)	0.75
Duration of symptoms before hospital admission, d, mean (SD)	6.7 (3.8)	6.6 (3.5)	0.99

*Statistical analyses used included Fisher exact test, χ^2^ test, and Student t test. SD, standard deviation; COPD, chronic obstructive pulmonary disease; PaO_2_/FiO_2_, partial pressure arterial oxygen and fraction of inspired oxygen ratio; CPK, creatine phosphokinase; U/L, units per liter. †Data available for 100 patients ‡Immunosuppressed patients include patients with autoimmune disorders (4 cases; one of them with renal transplantation), HIV infection (3 cases), lymphoma (1 case), and chronic renal failure (1 case). §Other disorders include cardiopathy (5 cases), Down syndrome (3 cases; 1 with cardiopathy), hypothyroidism (3 cases), pregnancy (1 case), and obstructive sleep apnea syndrome (1 case) ¶Data available for 98 patients #Data available for 93 patients **Data available for 91 patients

**Figure 1:**
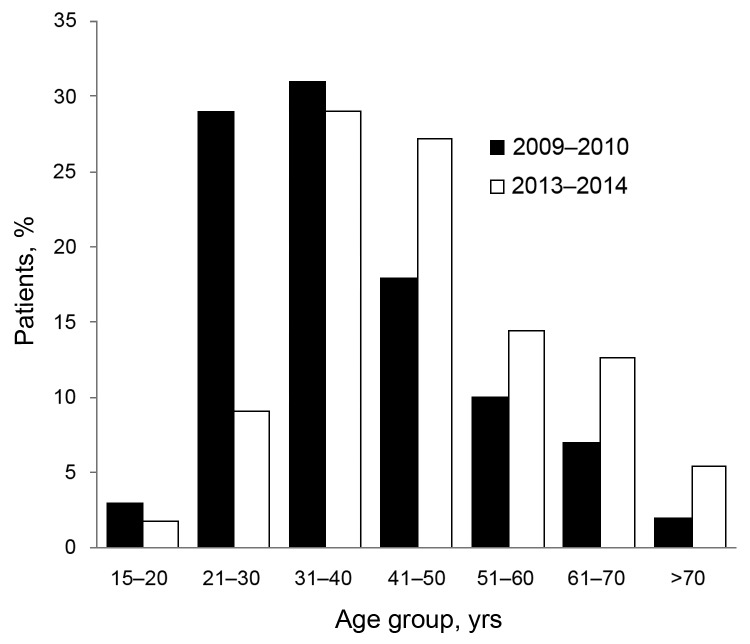
Patients with confirmed influenza pneumonia admitted to “Dr Ignacio Morones Prieto” (Hospital Central), in San Luis Potosí, Mexico during 2009–10 and 2013–14, according to age group.

To assess severity of illness and possible associated factors, we compared characteristics of the 55 patients with confirmed influenza A infection and 100 patients with A(H1N1)pdm09 infection admitted during the 2009–10 pandemic (Table 2). Patients in the 2013–14 cohort were somewhat older and heavier than those in the 2009–10 cohort: mean age of patients admitted during 2009–10 was 38.9 y and that of patients admitted during 2013–14 was 46.1 y (p = 0.002; Figure 1). Mean BMI of the 2009–10 cohort was 28.9, versus 31.9 for the 2013–14 cohort (p<0.001). Mean partial pressure arterial oxygen and fraction of inspired oxygen ratio was not significantly different for the 2 groups; however, proportion of those needing MV was significantly higher in the 2013–14 cohort (36/55 [65.5%] than in the 2009–10 cohort (47/100 [47%];p = 0.03).

**Table 2:** Demographic features of adult patients admitted with confirmed influenza virus pneumonia during December 5, 2013–February 7, 2014 compared with patients with confirmed influenza A(H1N1)pdm09 infection during the 2009–10 pandemic period, San Luis Potosí, Mexico*

Characteristics	2009–10, n = 100	2013–14, n = 55	p value
Age, y, mean (SD) median (range)	38.9 (13.1), 37 (15–71)	46.1 (14), 45 (19–79)	0.002
Sex, no. (%)			
M	52 (52)	28 (50.9)	0.89
F	48 (48)	27 (49.1)	
Presence of ≥1 underlying conditions, no. (%)	68 (68)	47 (85.5)	0.017
Obesity (body mass index>30)†	39 (39.8)	40 (74.1)	<0.001
Diabetes mellitus	20 (20)	15 (27.3)	0.3
Immunosuppression	8 (8) ‡	3 (5.5) §	0.75
Asthma-COPD-bronchiectasis	9 (9)	2 (3.6)	0.33
Other	19 (19)	4 (7.3) #	0.049
Any underlying disorder excluding obesity, no. (%)	45 (45)	23 (41.8)	0.7
Influenza vaccination during most recent season, no. (%)**	4 (4)	3 (5.5)	0.7
Clinical findings			
Body mass index, mean (SD) †	28.9 (5.9)	31.9 (3.9)	<0.001
PaO2/FiO2, mean (SD) ††	152.1 (102.5)	156.2 (69.3)	0.32
Total WBCs, K**/**μL, mean (SD) †	8.4 (5.3)	6.4 (3.2)	0.02
Lymphocytes, K**/**μL, mean (SD) ‡‡	0.94 (0.5)	0.85 (0.72)	0.37
C-reactive protein, mg/dL, mean (SD) § §	14.1 (9.8)	17.9 (10.9)	0.03
Lactate dehydrogenase, U/L, mean (SD) ¶¶	1,099.4 (769.6)	581.8 (387.9)	<0.001
Creatine phosphokinase, U/L, mean (SD) ##	530 (820.6)	480.6 (513.9)	0.69
Duration of symptoms before dyspnea, days, mean (SD) ***	2.1 (3.5)	3 (3.4)	0.13
Dyspnea duration before admission, days, mean (SD) †††	2.7 (2.7)	3.8 (2.6)	0.03
Duration of symptoms before hospital admission, days, mean (SD) **	4.7 (6.6)	6.6 (3.8)	0.003
Other features			
Requirement for mechanical ventilation, no. (%)	47 (47)	36 (65.5)	0.03
Death within 5 d from admission, no. (%)	6 (6)	6 (10.9)	0.35

*Statistical analyses used included Fisher exact test, chi-square test, Student t test, and Mann Whitney u test; S/D, standard deviation; COPD, chronic obstructive pulmonary disease; PaO2/FiO2, partial pressure arterial oxygen and fraction of inspired oxygen ratio; U/L, units per liter.  † Data was available for 152 patients. ‡ Includes chronic renal failure (4 cases), chronic steroid use (3 cases), and leukemia (1 case). § HIV infection (1 case), autoimmune disorder (2 cases; 1 had renal transplant), ¶ Some patients had >1 underlying condition: heart failure (6 cases), pregnancy/puerperium (6 cases), hypothyroidism (4 cases), seizure disorder (2 cases), ischemic cardiopathy (1 case), microcephaly (1 case), Down syndrome (1 case), obstructive sleep apnea (1 case). # Down syndrome (2 cases); hypothyroidism (1 case), cardiopathy (1 case). ** Data available for 154 patients. †† Data available for 147 patients. ‡‡ Data available for 151 patients. § § Data available for 144 patients. ¶¶ Data available for 135 patients. ## Data available for 131 patients. *** Data available for 139 patients ††† Data available for 138 patients

**Figure 2:**
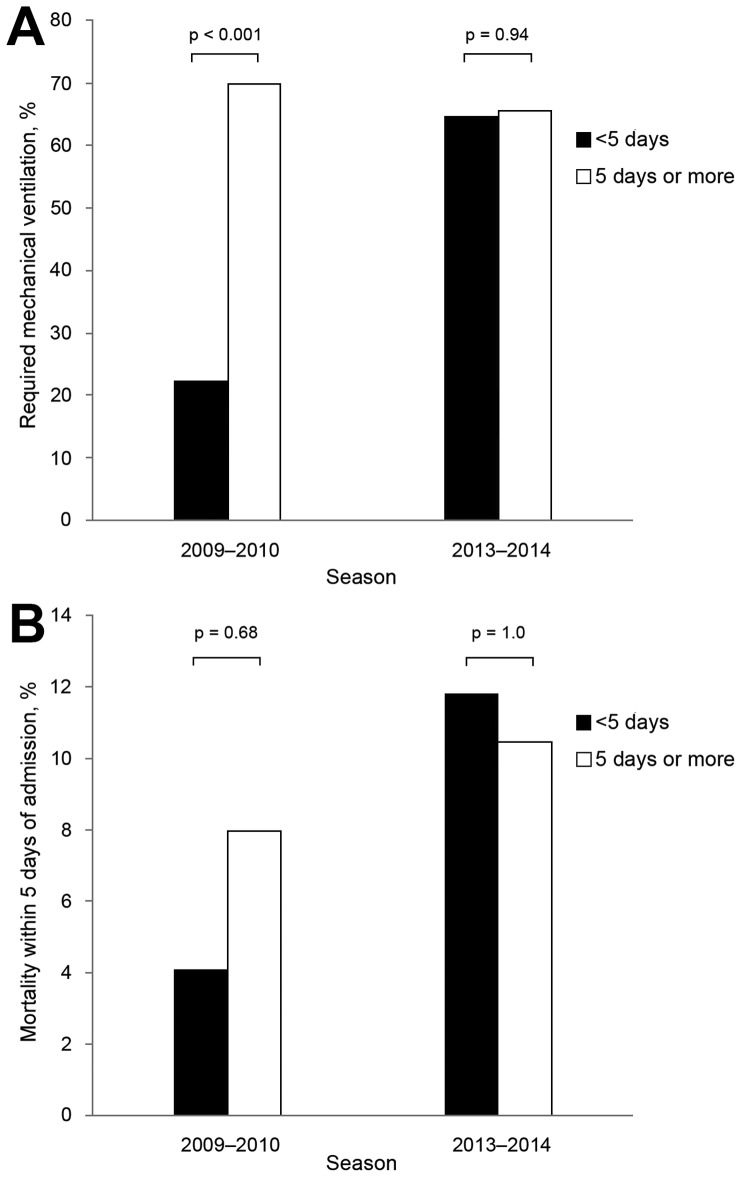
A) Proportion of patients with confirmed influenza pneumonia that required mechanical ventilation according to the duration of symptoms on admission (0–4 days versus >5 days) during the 2009–10 pandemic and 2013–14 season. B) Mortality rate within 5 days of admission according to the duration of symptoms on admission during the 2009–10 pandemic and 2013–14.

By the time of this report, 29 of the 102 patients admitted during 2013–14 had died, 69 had been discharged, and 4 remained hospitalized. Of 29 deaths, 11 (37.9%) occurred within 5 days of admission. The rate of deaths within 5 days of admission was 10.9% among patients with confirmed influenza infection admitted during 2013–14, and 6% among those admitted during 2009–10 (p = 0.35). In contrast to findings with the 2009–10 cohort, duration of symptoms among the 2013–14 cohort at the time of admission did not appear to affect the outcome (Figure 2).

In Mexico, the first 3 influenza A(H1N1)pdm09 waves were characterized by a high proportion of hospitalizations among persons 5–59 years of age (*1*,*9*); in the fourth wave, the proportion of hospitalizations increased in young children and older adults (*2*). The 2013–14 influenza season data resemble those of the initial A(H1N1)pdm09 outbreak in the high number of severe cases among adults <80 years and, although this observation has been described in other pandemics (*10*), possible explanations for this epidemiologic pattern should be analyzed. The following characteristics of the 2013–14 influenza season cohort could explain this demographic situation: 1) low vaccination coverage of high-risk populations; 2) differences in the size of the high-risk susceptible population; 3) high prevalence of diabetes mellitus and obesity; 4) antigenic drift; and 5) unusually low environmental temperatures during the 2013–14 winter season. The severity of pneumonia cases among adults <80 years of age during the 2013–14 influenza season, characterized by MV requirement, low lymphocyte counts, and high C-reactive protein and lactate dehydrogenase concentrations, could be explained by a high prevalence of concurrent health conditions (especially obesity), delay in health care access, or a change in virulence of the current influenza strain.

## Conclusions

Because of the high number of unvaccinated patients in this study cohort, the need to modify the intended population for influenza vaccination policies should be assessed. The vaccination rate was substantially lower among patients who required MV compared with those who did not; the overall vaccination rate among hospitalized patients contrasts with the high vaccination rates reported recently in Mexico for high-risk groups (71.7%–101.9%) (*11*,*12*). The association between severe influenza infection and obesity has been increasingly documented since the 2009 pandemic (*13*). Therefore, this group should be included among persons at high risk for influenza complications and the BMI range of subjects that require vaccination should include not only those with morbid obesity, but also those weighing >30 kg/m^2^.

To improve outcomes, additional measures could be implemented such as campaigns and educational programs for the public and physicians that advocate early diagnosis, treatment, and identification of influenza-associated complications. In summary, to establish appropriate preventive measures, the epidemiologic characteristics of influenza outbreaks should be used to identify risk factors for severe infections.
